# Idiopathic Gingival Fibromatosis: Case Report and Its Management

**DOI:** 10.1155/2009/153603

**Published:** 2010-03-10

**Authors:** Prashant P. Jaju, Ankit Desai, Rajiv S. Desai, Sushma P. Jaju

**Affiliations:** ^1^Department of Oral Medicine, Diagnosis and Radiology, MGV's KBH Dental College & Hospital, Maharashtra, Nashik 422003, India; ^2^Department of Periodontics, Terna Dental College, Mumbai 400706, India; ^3^Department of Oral Maxillofacial Pathology, Dr. D. Y. Patil Dental College and Hospital, Mahesh Nagar, Pimpri, Pune 411018, India; ^4^Dentocare multispeciality Clinic, Nashik Road, Nashik, Maharashtra 422101, India

## Abstract

Idiopathic gingival fibromatosis is a rare condition. We present a case of idiopathic gingival fibromatosis with its multidisciplinary approach of management. The clinical, radiographic, and histopathological features have been described in detail.

## 1. Introduction

Gingival hyperplasia is a bizarre condition causing esthetic, functional, psychological, and masticatory disturbance of the oral cavity. Causes of gingival enlargement can be due to plaque accumulation, due to poor oral hygiene, inadequate nutrition, or systemic hormonal stimulation [1]. Gingival enlargements are also pragmatic in several blood dyscrasias such as leukaemia, thrombocytopenia, or thrombocytopathy. A progressive fibrous enlargement of the gingiva is a facet of idiopathic fibrous hyperplasia of the gingival [2]. Idiopathic gingival fibromatosis is a rare hereditary condition that has no definite cause [3]. Investigations are in evolution to establish the genetic linkage and heterogeneity associated with it [4, 5]. This condition may manifest as an autosomal dominant or, less commonly, an autosomal recessive mode of inheritance, either as an isolated disorder or as part of a syndrome [6–9]. Autosomal-dominant forms of gingival fibromatosis, which are usually nonsyndromic, have been genetically linked to the chromosome 2p21-p222 and 5q13-q22.

In modern times, a mutation in the son of sevenless-1 (*SOS-1*) gene has been suggested as a possible cause of isolated (nonsyndromic) gingival fibromatosis, but no definite linkage has been established [10]. Idiopathic gingival fibromatosis is a gradually progressive benign enlargement that affects the marginal gingiva, attached gingival, and interdental papilla. The fibromatosis may potentially cover the exposed tooth surfaces, thereby hampering the functioning of the stomatognathic system. The gingival tissues are usually pink and nonhemorrhagic and have a firm, fibrotic consistency. Histopathologically, the bulbous increased connective tissue is relatively avascular, and has densely arranged collagen-fibre bundles, numerous fibroblasts, and mild chronic inflammatory cells. The overlying epithelium is thickened and acanthotic, and has elongated rete ridges [11–15]. The autosomal dominant form is often associated with hypertrichosis, corneal dystrophy, nail defects, deafness, and craniofacial deformities. In children suffering from autosomal dominant form, they may suffer from mental retardation and epilepsy. Autosomal recessive form facial anomalies with hypertelorism, have been observed but most forms are without defects, other than gingival enlargement. Consanguinity has been observed in recessive form. We report a case of nonsyndromic case of idiopathic gingival fibromatosis along with its management.

## 2. Case Report

**Figure 1:**
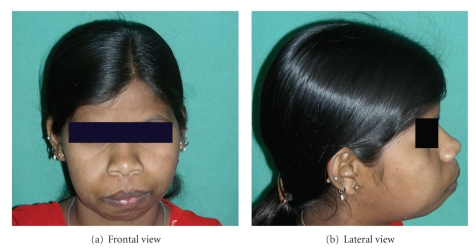
Preoperative photograph with fullness of face.

A-18-year-old unmarried poor Muslim girl reported to the outpatient department of Dr. DY Patil Dental College and Hospital, Pune, India with a chief complaint of swollen gums since she was 6 years old. (Figure 1) According to patient, swelling appeared at the time of eruption of permanent teeth. Swelling was not associated with pain. She reported to the department as she was having functional and masticatory difficulty. Patient's medical, dental, personal, and family histories were noncontributory. There was no history of consanguious marriage. Extraneoral examination showed facial disfigurement with protruding lips.

**Figure 2:**
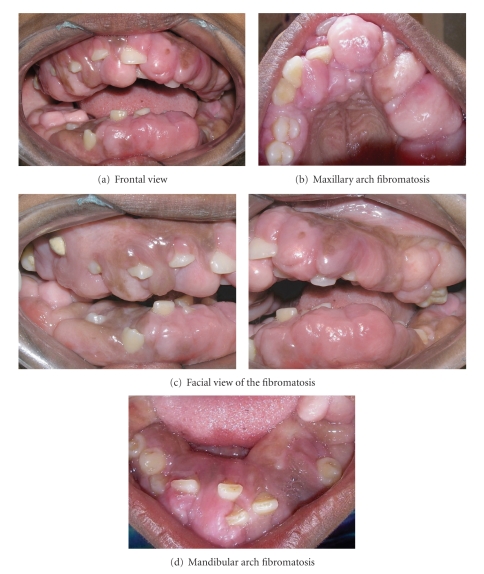
Pre-perative intraoral picture of generalized gingival fibromatosis.

Intraoral examination revealed enlargement of gingiva on both buccal and lingual/palatal sides with pinkish red in color, fibrous in consistency with absence of stippling. (Figure 2) Bleeding on probing was absent. Gingival enlargement enclosed the major surface of the teeth present except the incisal/occlusal surfaces. Clinical examination revealed upper right second molar, upper and lower left first and second molars, and lower right second premolar; first and second molars were absent. Mobility was seen in all the teeth present. There was increase in intermaxillary rest position. Based on the above findings, a provisional diagnosis of idiopathic generalized gingival fibromatosis was made. Intraoral periapical radiographs, panoramic radiograph, and lateral cephalogram radiograph were advised. Also a whole body general body examination and blood investigations were advised to eliminate any medical abnormalities.

**Figure 3:**
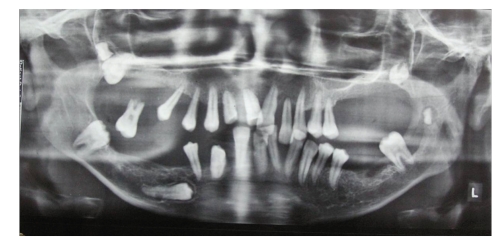
Panoramic radiograph showing generalized bone loss with floating teeth appearance.

**Figure 4:**
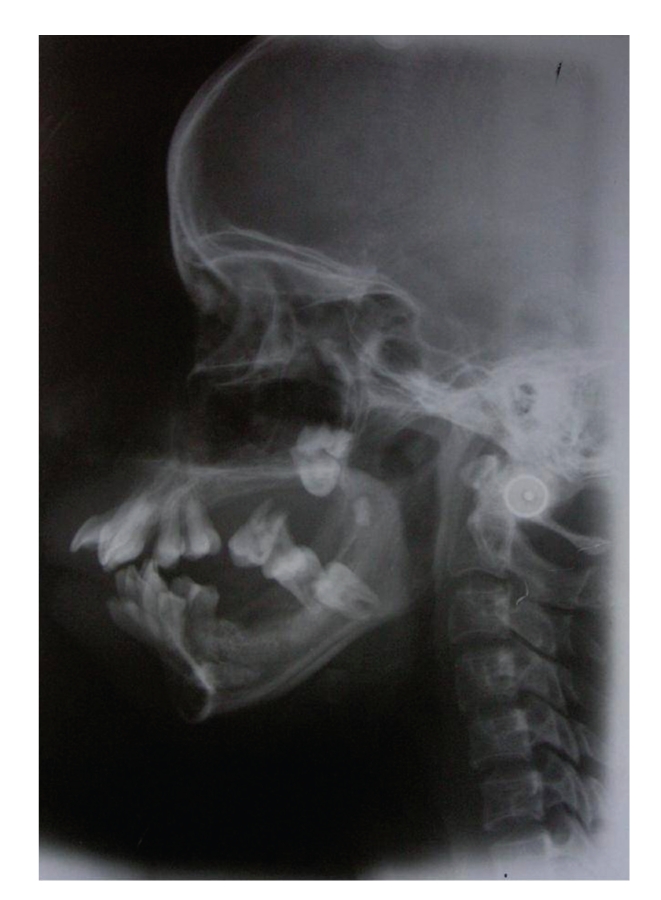
Lateral cephalogram showing the soft-tissue profile.

Radiographic examination revealed generalised bone loss giving floating teeth appearance in the region of all posterior teeth. There was displacement of upper second and third molars tooth bud in the tuberosity region. Also lower left second molar tooth bud was displaced in midramus region. Horizontally impacted lower right second premolar was seen. (Figures 3 and 4) Multidisciplinary approach for treatment was considered. Patient was referred to the periodontics department for further treatment. Treatment decided was full-mouth gingivectomy, extraction of mobile teeth, and prosthetic rehabilitation with upper and lower partial denture. Patient was educated about the impacted tooth which was planned to be extracted on being symptomatic.

### 2.1. Surgery

**Figure 5:**
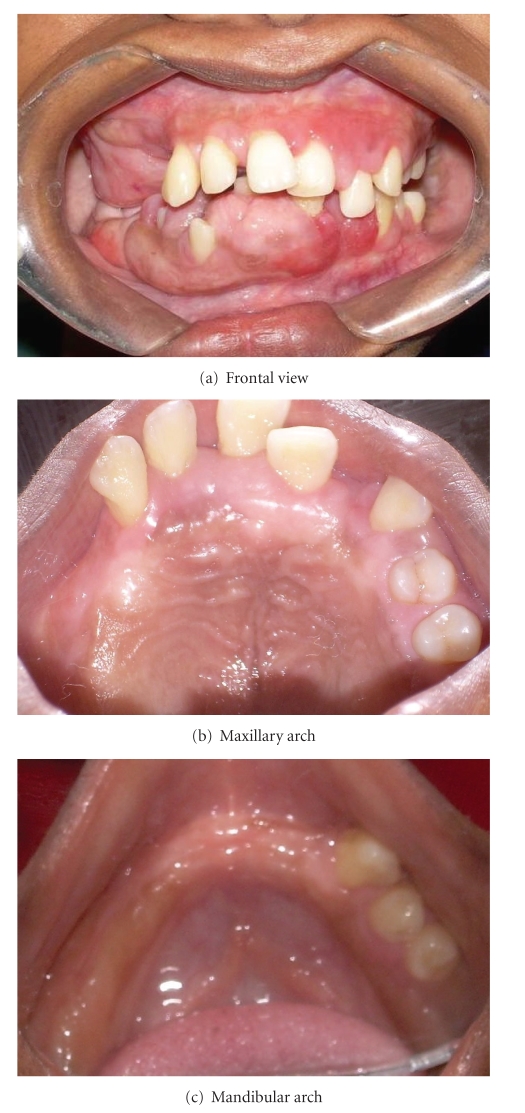
Postoperative intraoral view following gingivectomy.

**Figure 6:**
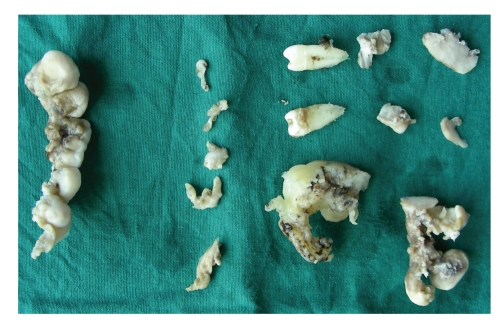
Excised tissue mass.

Considering the size and extent of gingival enlargement, a quadrant-wise gingivectomy was performed under local anaesthesia. An external bevel gingivectomy was done in all four quadrants. Mobile teeth were extracted at the time of surgery. Healing was uneventful. The total masses of excised gingival tissue were sent for histopathological examination (Figures 5 and 6).

### 2.2. Histopathological Report

**Figure 7:**
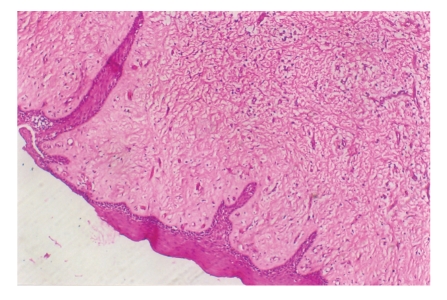
Histopathological picture.

**Figure 8:**
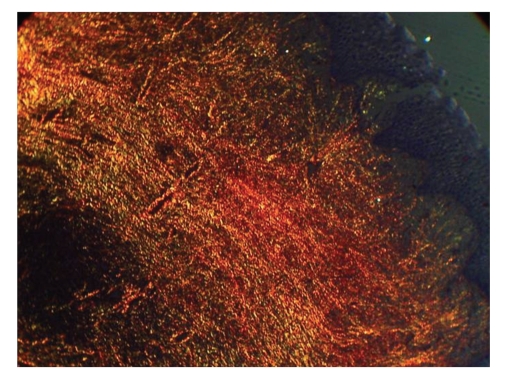
Picrosirus stain.

 Histopathological examination revealed abundance of collagen. Fibroblasts were increased in number, and various degrees of chronic inflammation were seen. The overlying epithelium exhibited some hyperplasia. Polarized microscope with picro sirus stain revealed more of mature collagen which stained red, while there was presence of immature collagen as well which stained green (Figures 7 and 8).

### 2.3. Prosthetic Stage

**Figure 9:**
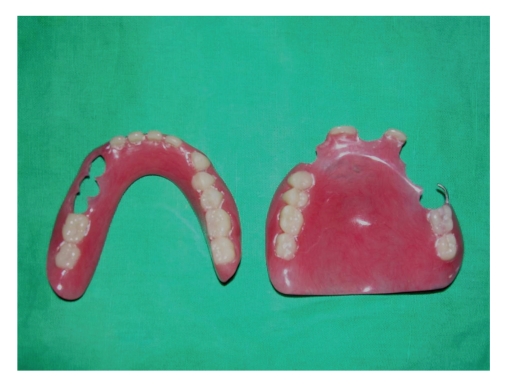
Partial denture constructed.

**Figure 10:**
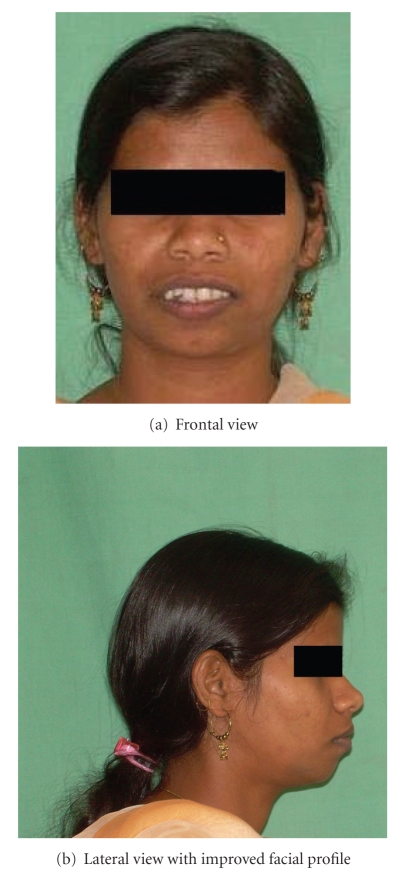
Final postoperative view with denture.

Following complete healing, a temporary partial denture was made. On completion of proposed treatment plan, patient's functional and aesthetic requirement improved significantly (Figures 9 and 10). Patient was very happy with the partial denture and refused any further treatment.

## 3. Discussion

Gingival overgrowth varies from mild enlargement of an isolated interdental papillae to segmental or uniform and marked enlargement affecting one or both of the jaws [16]. Here we reported a case of nonsyndromic idiopathic generalized gingival fibromatosis with its prosthetic rehabilitation by multidisciplinary approach. As the family, medical, prenatal, drug histories were noncontributory, we gave a diagnosis of idiopathic gingival fibromatosis. There are multiple causes of generalized gingival fibromatosis. (Table 1) Diverse syndromes having varying mode of inheritance are reported of having generalized gingival fibromatosis. (Table 2) The precise mechanism of idiopathic gingival fibromatosis is unknown but it appears to confine to the fibroblasts which harbor in the gingivae. The hyperplastic response does not involve the periodontal ligament and occurs peripheral to the alveolar bone within attached gingival [17]. The growth is linked with eruption of teeth as seen in present case, and the presence of teeth may be necessary for the commencement of the process. Fibromatosis gingivae may hinder tooth eruption, mastication, and oral hygiene. In severe cases, noneruption of the primary or permanent teeth may be the chief complaint of the patient [18]. Our patient suffered from difficulty in mastication and swallowing due to gingival overgrowth which resulted in atypical swallowing pattern [19]. Also due to the facial disfigurement, the family were worried about marriage prospect of the patient. The finest and suggested treatment modality for idiopathic gingival fibromatosis is gingivectomy [20]. Literature reports high recurrence rate after surgery, and needs a close follow up. Present case has been followed for 2 years with no recurrence. There is debate regarding the time of surgery. Eruption of complete set of permanent teeth is the recommended time for surgery [21]. Our patient had eruption of permanent teeth, and hence surgery was considered as the best approach. Histopathologically, fibromatosis shows a bulbous increase in the connective tissue which is relatively avascular and has densely arranged collagen-fibre bundles, numerous fibroblasts, and mild chronic inflammatory cells. The overlying epithelium is thickened and acanthotic and has elongated rete ridges. Our case report showed similar histological findings. Unusual findings include the presence of small calcified particles, amyloid deposits, islands of odontogenic epithelium, and osseous metaplasia in the connective tissue. Patient was advised to maintain good oral hygiene to minimize the effect of inflammation on fibroblasts.

## 4. Conclusion

Present case was of nonsyndromic idiopathic gingival enlargement, with its multidisciplinary management. Treatment was complete excision and prosthetic rehabilitation which appreciably improved the aesthetic and masticatory competence.

## Figures and Tables

**Table 1:** Causes of generalised gingival fibromatosis.

Hyperplastic gingivitis
Mouth-breathing gingivitis
Drug-induced gingival overgrowth
Scurvy
Gingival overgrowth in pregnancy
Gingival overgrowth due to leukemia
Hereditary gingival fibromatosis
Wegener granulomatosis
Acanthosis nigricans
Idiopathic variety

**Table 2:** Syndromes associated with gingival fibromatosis.

Syndrome	Clinical features	Mode of inheritance
Laband syndrome	Syndactily, nose and ear abnormalities, hyperplasia of the nails, and terminal phalanges	Dominant
Rutherfurd syndrome	Corneal dystrophy	Dominant
Cross syndrome	Microphthalmia, mental retardation, and pigmentary defects	Recessive
Ramon syndrome	Hypertrichosis, mental retardation, delayed development epilepsy, and cherubism	Recessive
